# The burden of carbapenem-resistant *Enterobacterales* infection in a large Thai tertiary care hospital

**DOI:** 10.3389/fphar.2022.972900

**Published:** 2022-09-02

**Authors:** Watcharaphon Tangsawad, Chayanis Kositamongkol, Piriyaporn Chongtrakool, Pochamana Phisalprapa, Anupop Jitmuang

**Affiliations:** ^1^ Department of Medicine, Faculty of Medicine Siriraj Hospital, Mahidol University, Bangkok, Thailand; ^2^ Department of Microbiology, Faculty of Medicine Siriraj Hospital, Mahidol University, Bangkok, Thailand

**Keywords:** hospital cost, mortality, burdens, carbapenem-resistant, Enterobacterales

## Abstract

**Background:** Carbapenem-resistant *Enterobacterales* (CRE) are resistant to several other classes of antimicrobials, reducing treatment options and increasing mortality. We studied the clinical characteristics and burden of hospitalized adult patients with CRE infections in a setting where treatment options are limited.

**Methods:** A retrospective cohort study included adult inpatients between January 2015–December 2019 at Siriraj Hospital in Bangkok, Thailand. Clinical and microbiological data were reviewed.

R**esults**: Of 420 patients with CRE infections, the mean age was 65.00 ± 18.89 years, 192 (45.72%) were male, and 112 (26.90%) were critically ill. Three hundred and eighty (90.48%) had *Klebsiella pneumoniae*, and 40 (9.52%) had *Escherichia coli* infections. The mean APACHE II score was 14.27 ± 6.36. Nearly half had previous hospitalizations (48.81%), 41.2% received antimicrobials, and 88.1% had undergone medical procedures before the onset of infection. The median time of onset of CRE infection was 16 days after admission. Common sites of infection were bacteremia (53.90%) and pneumonia (45.47%). Most CRE-infected patients had septic shock (63.10%) and Gram-negative co-infections (62.85%). Colistin (29.95%) and non-colistin (12.91%) monotherapies, and colistin-based (44.78%) and non-colistin-based (12.36%) combination therapies were the best available antimicrobial therapies (BAAT). The median length of hospitalization was 31 days, and the median hospitalization cost was US$10,435. The in-hospital mortality rate was 68.33%. Septic shock [adjusted odds ratio (aOR) 10.73, 5.65–20.42, *p* <0 .001], coinfection (aOR 2.43, 1.32–4.47, *p* = 0.004), mechanical ventilation (aOR 2.33, 1.24–4.36, *p* = 0.009), and a high SOFA score at onset (aOR 1.18, 1.07–1.30, *p* <0 .001) were associated with mortality.

**Conclusion:** CRE infection increases mortality, hospital stays, and healthcare costs. A colistin-based regimen was the BAAT in this study. Therefore, newer antimicrobial agents are urgently needed.

## Introduction

The prevalence of Carbapenem-resistant *Enterobacterales* (CRE) infection varies by geographic region. For example, in China, CRE occurs at a rate of 0.32–14.38/100,000 patient-days (Zhang et al., 2018) compared with 0.3–2.93/100,000 patient-days in the U.S. (Livorsi et al., 2018). In Thailand, CRE infections have been rapidly increasing from 3.37/100,000 patient-days in 2011 to 32.49/100,000 patient-days in 2016 (Chotiprasitsakul et al., 2019). At Siriraj Hospital, nearly half of patients who developed hospital-associated infections were infected by multidrug-resistant Gram-negative bacteria (Chaisathaphol and Chayakulkeeree, 2014). *Klebsiella pneumoniae* and *Escherichia coli* are common species of *Enterobacterales* that are evolving carbapenem resistance (de Maio Carrilho et al., 2016; Wang et al., 2016; McConville et al., 2017; Zhang et al., 2018). The rate of CRE isolation varies widely based on the types of clinical samples. In our institute, 2.1% of *E. coli* and 24.7% of *K. pneumoniae* were isolated from urine (Sirijatuphat et al., 2020), 23.0% of *K. pneumoniae* were isolated from the respiratory tract system (Jitmuang et al., 2020), and 1.3% of *E. coli* and 20.0% of *K. pneumoniae* were isolated from blood samples (Sirijatuphat et al., 2018). Solid organ transplant, chronic kidney disease, mechanical ventilatory support, urinary or central venous catheterization placement, tracheostomy, and prolonged hospitalization are associated with CRE infection (Livorsi et al., 2018; Zhang et al., 2018; Kang et al., 2019). Previous administration of broad-spectrum antimicrobials, such as third- or fourth-generation cephalosporins, carbapenems, and beta-lactam/beta-lactamase inhibitors, are also associated with CRE infection (Livorsi et al., 2018; Zhang et al., 2018; Kang et al., 2019). CRE are also resistant to several other classes of antimicrobials, limiting treatment options. However, they remain susceptible *in vitro* to conventional antimicrobials such as colistin, polymyxin B, tigecycline, aminoglycosides, and fosfomycin (de Maio Carrilho et al., 2016; Zhang et al., 2018; Chotiprasitsakul et al., 2019). CRE isolates have a higher susceptibility rate to colistin, tigecycline, and aminoglycosides (Szekely et al., 2021), regarded as the best available treatment options, whereas fosfomycin exhibits moderate susceptibility (Leelawattanachai et al., 2020). CRE infection can result in a prolonged length of stay (Chaisathaphol and Chayakulkeeree, 2014; Kang et al., 2019), substantial increases in hospital costs (Lavagnoli et al., 2017), and increased mortality (Wang et al., 2016; McConville et al., 2017; Zhang et al., 2018; Chotiprasitsakul et al., 2019).

In Thailand, little is known about CRE infection’s clinical and healthcare burden. Colistin-based antimicrobial therapy is the most common regimen to treat this infection, but the treatment effectiveness of other best-available antimicrobial agents has not been reported. Therefore, we aimed to describe the clinical characteristics and healthcare burden of CRE infection in a setting where treatment options are limited and to compare the outcomes among different best-available antimicrobial therapy (BAAT) administered to CRE-infected patients.

## Materials and methods

We conducted a retrospective cohort study by reviewing the medical charts of adult patients hospitalized from January 2015 to December 2019. In addition, patient clinical characteristics, microbiological data, and treatment outcomes were collected. The Scientific Ethics Committee approved the study’s protocol, Siriraj Institutional Review Board (SIRB) (Approval no. Si 242/2020). In addition, the requirement for informed written consent from patients was waived because of the retrospective chart reviews.

### Study populations

Patients over 18 years of age with clinical signs and at least one positive culture from any specimen that indicated CRE infection were included. A positive culture without evidence of clinical infection was considered to be colonization and was excluded from the analysis. The terms “infection” and “colonization” were defined according to the United States Centers for Disease Control and Prevention (US CDC) definitions for nosocomial infection surveillance (Horan et al., 2008). “Infection” was defined as clinical symptoms and signs, including laboratory and/or radiological findings suggestive of a specific organ of infection. Meanwhile, “colonization” was defined as the presence of microorganisms in patient samples, but they were not causing adverse clinical symptoms and signs of infection. When patients had multiple episodes of CRE infection, only the first infection episode was analyzed.

### Data collection

A microbiological dataset of all clinical samples with identified CRE isolates between study periods was retrieved and verified by a clinical microbiologist, Department of Microbiology. Study investigators were responsible for reviewing the microbiological dataset with matching clinical data to select CRE-infected patients suitable for further analysis. Samples with CRE colonization, samples from pediatric patients, and duplicate samples from the same patient were excluded from the analysis. The medical record of each patient was reviewed to obtain demographic data, hospitalization unit, comorbidities, previous antimicrobial therapy, previous chemotherapy, recent operations or medical procedures, catheterization, duration of hospitalization, type of infection attributed to CRE infections, co-infection with other organisms, treatments, outcomes of the CRE infections, all-cause in-hospital mortality, and hospital costs. In addition, microbiological data were collected, including the source and type of the clinical sample, co-pathogens isolated from the sample, and the antimicrobial susceptibilities of the CRE isolates. The study data were recorded in the case record form before the final analysis.

A conventional Gram-negative biochemical testing panel was usually performed in our institute to identify Gram-negative bacterial isolates. The Clinical and Laboratory Standards Institute (CLSI) disk diffusion (D.D.) method was conducted for *Enterobacterales* antimicrobial susceptibility testing. We used the CLSI interpretative breakpoint criteria (CLSI 27th ed. M100S, 2017) to define a carbapenem-resistant isolate (CLSI, 2017). CRE was defined as an isolate resistant to one or more of the following carbapenems; ertapenem, imipenem, meropenem, or doripenem. We excluded some *Enterobacterales* isolates, such as *Proteus* spp., *Providencia* spp., and *Morganella* spp., because these organisms may have elevated minimal inhibitory concentrations (MICs) or intrinsic resistance to imipenem by non-carbapenemase producing mechanisms (Weinstein and Lewis, 2020). When a carbapenem-resistant isolate was identified, we performed colistin and fosfomycin susceptibility testing by the D.D. method. Based on our previous study, at colistin MICs breakpoints of 1 mg/L or less and 2 mg/L or less from the broth microdilution method, colistin inhibition zone diameters of ≤11 mm or ≥14 mm from the D.D. method were accurately predicted colistin susceptibility, whereas the inhibition zone diameters between 12–13 mm were not sufficiently correlated to predict susceptibility (Ruangkriengsin et al., 2018). Accordingly, our microbiology laboratory routinely reports the results as inhibition zone diameter for the colistin D.D. method without interpretation since there are still no recommended breakpoints relative to colistin susceptibility results from the D.D. method (Weinstein and Lewis, 2020). Meanwhile, the breakpoint criteria of the fosfomycin D.D. method are restricted to use for *E. coli* isolated from urine specimens only. Therefore, the inhibition zone diameter of the fosfomycin D.D. method is reported without interpretation when non–*E. coli* isolates or any non–urine isolates are identified.

### Definitions

Hospital-associated infection (HAI) was defined as an episode of infection that occurred more than 48 h after hospitalization. Community-acquired infection (CAI) was defined as an infection onset occurring at a patient’s home who had no recent contact with a healthcare facility or an onset of infection in the first 48 h of hospitalization. Immunosuppression is the administration of immunosuppressive therapy for an autoimmune or inflammatory disease, chemotherapy for neoplasia, or systemic corticosteroids ≥20 mg/day for at least 3 weeks, including the presence of leukemia, lymphoma, HIV infection, organ transplant recipient, or splenectomy. The source of CRE infection was determined by the anatomical location of the active infection where the CRE isolate was identified. Empiric antimicrobial therapy was defined as the antimicrobial agent(s) administered from the infection onset before knowing the final microbiological results. Appropriate empiric treatment was the timely administration of antimicrobial agents with *in vitro* activity against the causative isolate before knowing the microbiological results. Definitive antimicrobial therapy was defined as the antimicrobial agent(s) administered soon after the culture, and the antimicrobial susceptibility results were available. Best-available antimicrobial therapy (BAAT) was the most appropriate antimicrobial treatment that was considered the most active and best-available regimen to treat CRE infection. The primary outcome was all-cause in-hospital mortality, defined as death from any cause during hospitalization.

### Sample size calculation

There are several factors associated with CRE infection, including the use of antimicrobials during the preceding 3 months, ICU admission, invasive or surgical procedures, mechanical ventilatory support, placement of a central venous catheter, having diabetes mellitus, hemodialysis, having a solid tumor, being in an immunocompromised state or undergoing chemotherapy in the preceding 6 months, and having been diagnosed with chronic obstructive pulmonary disease (COPD) (Wang et al., 2016). The sample size was calculated by estimating the proportion of one group using the proportion of COPD (15%) as it was the least associated factor (Wang et al., 2016). Applying 25% precision to the COPD proportion in CRE infection equal to 0.375, alpha = 0.05, and power = 80%, the minimum sample size would be 358. We estimated that 10% of cases would have incomplete data and could not be included. Thus, the final required sample size was 400 subjects.

### Statistical analysis

Data were analyzed using IBM SPSS Statistics for Windows, version 20.0 (IBM Corp., Armonk, NY, United States). Univariable analysis was performed to explore factors associated with all-cause in-hospital mortality. The Student’s t-test was used to compare continuous variables, and the chi-squared or Fisher’s exact test was used for categorical variables. Any variable determined to have a significant association (*p*-value < 0.05) with mortality in the univariable analysis was subsequently entered into a multivariable, forward-stepwise logistic regression model. A two-tailed *p*-value of < 0.05 was considered statistically significant. The direct-medical costs of hospitalization were calculated based on medical billing charges of related IPD visits of all patients in the cohort. These data were retrieved from the hospital’s electronic database. The charges included in that medical billing comprised charges related to drugs, surgical procedures, anesthesia, laboratory investigations, medical devices, hospital rooms and services, physical therapies, and medical fees. The related charges were then converted to costs using a hospital-specific cost-to-charge ratio (cost-to-charge ratio = 1) (Phisalprapa et al., 2020). The admission costs in 2015–2019 were inflated to 2021 Thai Baht using Thai medical care consumer price index, as displayed in Supplementary Table S1. All cost data are presented as the median (interquartile range) in 2021 U.S. Dollar. The exchange rate was 31.98 Thai Baht per 1 U.S. Dollar.

## Results

### Clinical characteristics, treatment outcomes, and total hospital cost of patients with CRE infections

**FIGURE 1:**
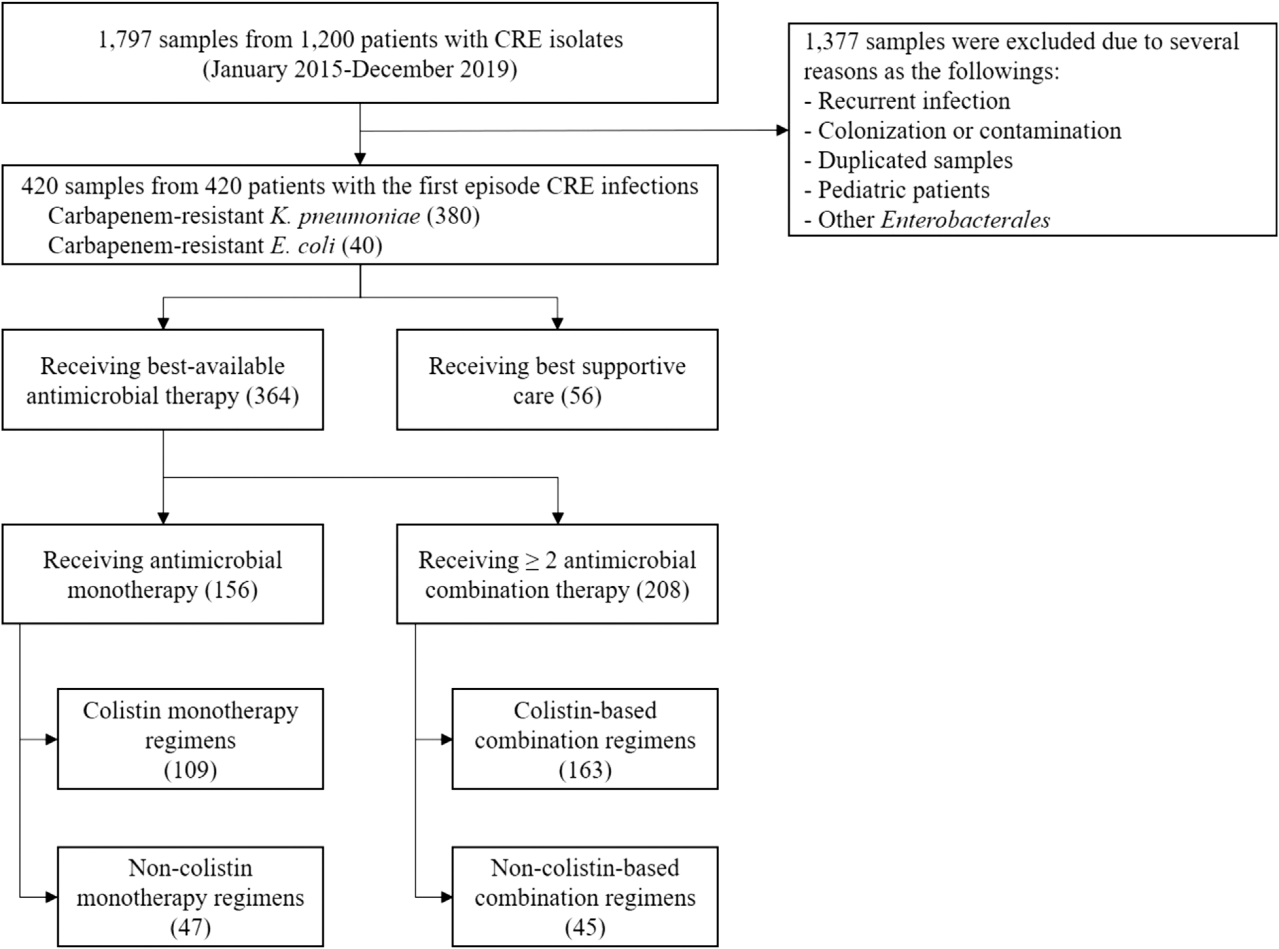
Study flow chart.

A total of 1,797 samples from 1,200 patients with CRE isolates were reviewed between January 2015 and December 2019. Of these, 420 adult hospitalized patients with CRE infections were included; 380 (90.48%) were carbapenem-resistant *K. pneumoniae* infections, and 40 (9.52%) were carbapenem-resistant *E. coli* infections. In addition, small proportions of other carbapenem-resistant *Enterobacterales* such as *Enterobacter* spp., *Citrobacter* spp., and *Serratia* spp. were identified, but we did not include these isolates in this study (Figure 1). The mean age of patients was 65.06 ± 18.89 years; 192 (45.72%) were male. Several comorbidities, such as hypertension (54.76%), solid and hematologic malignancies (61.90%), diabetes mellitus (34.28%), chronic kidney disease (24.76%), cardiac diseases (19.05%), and liver diseases (16.43%) were found (Table 1). Most patients were admitted to medicine wards (55%) or the intensive care unit (ICU) (26.90%). Infectious diseases (61.20%) were the main reason for the hospitalization of these patients. At admission, the mean Charlson comorbidity index was 4.87 ± 2.60, and the mean Acute Physiological Assessment and Chronic Health Evaluation (APACHE) II score was 14.27 ± 6.36. Nearly half of the patients with CRE infections had been hospitalized (48.81%) or had received antimicrobial therapy (41.20%) in the preceding 3 months, whereas only 9.52% had prior colonization with CRE isolates (Table 1). During admission, 178 patients received 214 antimicrobial prescriptions before the onset of CRE infection. Carbapenem (38.79%), piperacillin-tazobactam (21.02%), and cephalosporins (18.69%) were the most common agents administered. Most patients underwent therapeutic medical procedures prior to the onset of CRE infection, including mechanical ventilatory (MV) support (62.86%), placement of central venous catheterization (CVC) (49%), hemodialysis (H.D.) (32.14%), and major surgery (24.29%) (Table 1).

**TABLE 1:** Baseline characteristics, clinical data, management, treatment outcomes, and hospitalization cost of patients with carbapenem-resistant *Enterobacterales* infection.

Characteristics	Total (*n* = 420)
Male, *n* (%)	192 (45.72)
Female, *n* (%)	228 (54.28)
Age, mean ± S.D., years	65.06 ± 18.89
Body mass index, mean ± S.D., kg/m^2^	22.89 ± 5.11
Underlying diseases, *n* (%)	
Hypertension	230 (54.76)
Solid malignancy	183 (43.57)
Diabetes mellitus	144 (34.28)
Chronic kidney disease	104 (24.76)
Cardiac diseases	80 (19.05)
Liver diseases	69 (16.43)
Hematologic malignancy	77 (18.33)
Cerebrovascular diseases	56 (13.33)
Receiving immunosuppressive agent	55 (13.01)
Autoimmune diseases	35 (8.33)
Chronic lung diseases	16 (3.81)
Post-organ transplantation	14 (3.33)
HIV infection	8 (1.90)
Ward/unit of hospitalization, *n* (%)	
Medicine	231 (55.00)
ICU	112 (26.90)
Surgery	68 (16.20)
Emergency unit	9 (2.14)
Cause of hospitalization, *n* (%)	
Infection-related	256 (61.20)
Non-infection related	164 (39.04)
Charlson comorbidity index, mean ± S.D.	4.87 ± 2.60
APACHE II score at admission, mean ± S.D.	14.27 ± 6.36
Past medical history, *n* (%)	
Previous antimicrobial administration in the last 3 months	173 (41.20)
Previous hospitalization in last 3 months	205 (48.81)
Prior CRE colonization^a^	40 (9.52)
Previous surgery within 1 month	18 (4.29)
Type of previous antimicrobial administration^b^, *n* (%)	
Carbapenems	83 (38.79)
Piperacillin-tazobactam	45 (21.02)
Cephalosporins	40 (18.69)
Quinolones	23 (10.75)
Vancomycin	12 (5.61)
Amoxi-clavulanate	5 (2.34)
Aminoglycosides	5 (2.34)
Colistin	1 (0.47)
Medical procedure before the onset of CRE infection, *n* (%)	
On mechanical ventilatory support	264 (62.86)
Central venous catheter placement	206 (49.00)
On hemodialysis	135 (32.14)
Major surgery	102 (24.29)
Receiving TPN	62 (14.76)
Receiving concurrent immunosuppressants	55 (13.01)
Tracheostomy	47 (11.20)
Percutaneous intervention	46 (10.92)
Concurrent chemotherapy	39 (9.29)
Bronchoscopy	37 (8.81)
ERCP	34 (8.07)
EGD	26 (6.18)
Colonoscopy	17 (4.21)
Minor surgery	11 (2.61)
Clinical features of CRE infection	
Onset before developing CRE infection, median (IQR), days	16 (Ni et al., 2016; McConville et al., 2017)
Site of CRE infection, *n* (%)	
Bacteremia	138 (53.90)
Pneumonia	117 (45.47)
Intraabdominal infection	88 (34.38)
Urinary tract infection	55 (21.50)
Others	22 (8.60)
SOFA score at onset, mean ± S.D.	7.01 ± 4.09
Septic shock, *n* (%)	265 (63.10)
Co-infection with other bacteria, *n* (%)	268
*Acinetobacter baumannii*	116 (43.28)
*Pseudomonas aeruginosa*	85 (31.72)
*Enterobacter* spp.	54 (20.15)
*Stenotrophomonas maltophilia*	54 (20.15)
*Enterococcus* spp.	64 (23.88)
*Staphylococcus* spp.	26 (9.70)
Others	16 (5.97)
Antimicrobial administration, *n* (%)	364^c^
Appropriate empiric treatment	37 (10.16)
Inappropriate empiric treatment	327 (89.84)
Best available antimicrobial therapy (BAAT)	
Antimicrobial monotherapy, *n* (%)	156 (42.86)
Colistin monotherapy	109 (69.87)
Non-colistin monotherapy	47 (30.13)
Antimicrobial combination therapy, *n* (%)	208 (57.14)
Colistin-based combination therapy	163 (78.37)
Non-colistin-based combination therapy	45 (21.63)
Outcomes	
Death, *n* (%)	287 (68.33)
Length of hospitalization, median (IQR), days	31 (19, 56)
Hospitalization cost (US$)^d^	
The total cost of the whole cohort	6,469,907
Median hospitalization cost per admission/person (IQR)	10,435 (5,090, 20,554)
Best supportive care with no antimicrobial therapy, median (IQR)	7,683 (3,185, 13,844)

a Defined as culture positive but without clinical infection and no antimicrobial therapy.

b From 178 patients (214 antibiotics prescribed).

c Fifty-six patients received only supportive care without antimicrobial therapy.

d The exchange rate of 2021 was 31.98 Thai Baht/U.S. Dollar (Source: Bank of Thailand, https://www.bot.or.th/App/BTWS_STAT/statistics/ReportPage.aspx?reportID=123&language=th

APACHE II, acute physiological assessment and chronic health evaluation II; BAAT, best available antimicrobial therapy; CRE, carbapenem-resistant Enterobacterales; EGD, esophagogastroduodenoscopy; ERCP, endoscopic retrograde cholangiopancreatography; HIV, human immunodeficiency virus; SOFA, sequential organ failure assessment; TPN, total parenteral nutrition; US$, U.S. Dollar.

The median onset of CRE infection occurred on the 16th (IQR 6, 30) day of hospitalization. Bacteremia (53.9%) and pneumonia (45.47%) were the most common sites of CRE infection. The mean SOFA score at the onset of infection/symptoms was 7.01 ± 4.09, while 265 (63.10%) had septic shock (Table1). Two hundred and sixty-eight (63.80%) patients had co-infections with other bacteria, particularly *Acinetobacter baumannii* (43.28%) and *Pseudomonas aeruginosa* (31.72%). Appropriate empiric antimicrobial treatments for the CRE infection were administered in only 37 (10.16%) patients.

Of 420 patients with CRE infection, 364 (86.67%) received the BAAT, and 56 (13.33%) received only the best supportive care without antimicrobial therapy. Among patients who received the BAAT, 156 (42.86%) received colistin (69.87%) and non-colistin (30.13%) monotherapy, whereas 208 (57.14%) received ≥2 antimicrobials; colistin-based (78.37%) and non-colistin based (21.63%) therapy (Table 1 and Figure 1). The best available agents other than colistin that were administered as a single or combination regimen were fosfomycin (40%), tigecycline (3.68%), aminoglycosides (5.78%), and meropenem (3.42%). The median length of hospitalization was 31 (IQR 19, 56) days, and the all-cause in-hospital mortality rate was 68.33%. The median hospitalization cost per admission was US$10,435 (IQR 5,090, 20,554). The total cost of the entire cohort was US$6,469,9076.

### Clinical characteristics and management between survival and non-survival groups and factors associated with all-cause in-hospital mortality

To identify factors associated with all-cause in-hospital mortality in patients with CRE infection, we compared clinical variables between patients who did and did not survive (Tables 2 and Supplementary Table S2). We did not include 56 terminally ill patients who received only the best supportive care since our objective was to study treatment outcomes with the BAAT; thus, a total of 364 patients, including 232 non-surviving and 132 surviving patients, were analyzed (Supplementary Table S2). Patients with CRE infection in the non-survival group had several clinical characteristics that were significantly different from patients in the survival group. For example, patients who did not survive were older (68.16 versus 59.79 years, *p* <0 .001) and had a higher mean Charlson comorbidity index (5.11 vs. 4.34, *p* = 0.004) and higher mean APACHE II score (14.86 vs. 12.06, *p* < 0.001). Furthermore, more patients required MV support (*p* < 0.001), placement of CVC (*p* < 0.001), hemodialysis (*p* < 0.001), and total parenteral nutrition (*p* = 0.034) before the onset of CRE infection. The non-survival group also had a more extended period of hospitalization before the onset of CRE infection (19 vs. 11 days, *p* <0 .001). In addition, there was a greater incidence of pneumonia in the non-survival group (*p* < 0.001), and a higher rate of intra-abdominal and urinary tract infections was observed in the survival group (*p* < 0.001). CRE bacteremia was higher in the non-survival group but was not statistically significant (*p* = 0.493). Patients in the non-survival group had significantly higher median SOFA scores (8.00 vs. 4.00, *p* < 0.001), and most of those patients (83.73%) developed septic shock (*p* < 0.001) and had co-infections with other bacteria (74.47%) (*p* < 0.001).

**TABLE 2:** Factors associated with all-cause in-hospital mortality in patients who had carbapenem-resistant *Enterobacterales* infection.

Variable	Univariate		Multivariate	
	OR (95% CI)	*p*-value	OR (95% CI)	*p-*value
Age	1.02 (1.01–1.04)	<0.001		
On mechanical ventilatory support	7.31 (4.40–12.17)	<0.001	2.24 (1.24–4.36)	0.009
Central venous catheter placement	3.90 (2.41–6.34)	<0.001		
On hemodialysis	3.53 (2.03–6.28)	<0.001		
Onset of CRE infection	1.02 (1.01–1.03)	<0.001		
Charlson comorbidity index	1.12 (1.03–1.23)	<0.001		
APACHE II score at admission	1.08 (1.04–1.12)	<0.001		
SOFA score at onset	1.38 (1.27–1.50)	<0.001	1.18 (1.07–1.30)	<0.001
Septic shock	22.30 (12.05–41.78)	<0.001	10.74 (5.65–20.42)	<0.001
Co-infection with other bacteria	3.68 (2.28–5.96)	<0.001	2.43 (1.32–4.47)	0.004
Colistin containing regimen	1.92 (1.15–3.19)	0.008		

APACHE II, acute physiological assessment and chronic health evaluation II; CRE, carbapenem-resistant Enterobacterales; SOFA, sequential organ failure assessment.

The BAAT, which included both monotherapy and combination regimens, did not significantly differ in survival outcomes. However, among patients that received monotherapy regimens, non-colistin monotherapy had a significantly higher survival rate (*p* = 0.002). Among groups with combination regimens, non-colistin-based combination therapy had a significantly lower survival rate (*p* = 0.002) (Supplementary Table S2). Several factors were associated with in-hospital mortality in the univariable analysis (Table 2). However, only being on MV support (adjusted OR [aOR] 2.24; 95% CI 1.24–4.36, *p* = 0.009), having a high SOFA score at the onset of infection (aOR 1.18; 95% CI 1.07–1.30, *p* < 0.001), having septic shock (aOR 10.74, 95% CI 5.65–20.42, *p* < 0.001), and having co-infection with other organisms (aOR 2.43, 95% CI 1.32–4.47, *p* = 0.004) were significantly associated with in-hospital mortality in the multivariable logistic regression model. In a subgroup analysis, infections caused by carbapenem-resistant *K*. *pneumoniae* had more fatal outcomes than those caused by carbapenem-resistant *E. coli* (69.74% versus 45%, *p* = 0.057). However, there was no association between the type of CRE organism and in-hospital mortality (data not shown). In addition, the mortality of CRE-infected patients in general medical wards (61.8%) and surgical wards (38.5%) had a statistically significant difference (*p* < 0.01). Patients in the surgical wards had fewer numbers of CRE pneumonia, septicemia, and septic shock, including had a lower median SOFA score at the onset compared to patients in the medical wards (data not shown).

### Rate of antimicrobial susceptibility of carbapenem-resistant *Klebsiella pneumoniae* and *Escherichia coli* strains

Antibiotic susceptibility testing (AST) demonstrated that aminoglycosides such as amikacin, gentamicin, and netilmicin, exhibited the highest susceptibility rate, ranging from 31.9% to 97.5% (Table 3). Carbapenem-resistant *E. coli* had high susceptibility to amikacin (97.5%) and moderate susceptibility to netilmicin (72.97%), whereas carbapenem-resistant *K*. *pneumoniae* had low to moderate susceptibility to gentamicin (68.1%), amikacin (38.5%), and netilmicin (31.9%). In addition, approximately 21.0%–27.5% of *E. coli* isolates remained susceptible to Group 2 carbapenems, such as imipenem, meropenem, and doripenem. The mean inhibition zone diameters of colistin and fosfomycin by the D.D. method are displayed in Table 3. Of 305 *K*. *pneumoniae* isolates tested, the mean inhibition zone diameters of colistin and fosfomycin D.D. were 12.95 ± 2.87 mm and 12.50 ± 4.63 mm, respectively. Of 27 *E. coli* isolates tested, colistin and fosfomycin D.D.'s mean inhibition zone diameters were 14.77 ± 2.64 mm and 23.19 ± 5.04 mm, respectively.

**TABLE 3:** Rate of antimicrobial susceptibility (%) of carbapenem-resistant *Klebsiella pneumoniae* and *Escherichia coli* and number of isolates tested.

Antimicrobial agent	*K. pneumoniae*		*E. coli*	
	No. of isolates tested	Susceptibility (%)	No. of isolates tested	Susceptibility (%)
Amikacin	380	38.5	39	97.5
Amoxi-clavulanate	380	0	40	0
Cefepime	380	0.7	40	2.5
Cefotaxime	380	1.57	40	0
Cefoxitin	379	4.2	40	10
Ceftazidime	380	0.5	40	5
Ceftriaxone	380	0.2	40	0
Cefuroxime	380	14.2	40	0
Ciprofloxacin	380	1.8	40	0
Doripenem	351	2.0	38	21.1
Ertapenem	380	0.2	40	0
Gentamicin	380	68.1	40	42.5
Imipenem	380	3.9	40	27.5
Meropenem	380	2.3	40	22.5
Netilmicin	376	31.9	37	72.97
Piperacillin-tazobactam	380	0.2	40	7.5
Tetracycline	380	11.8	40	5
Cotrimoxazole	380	12.3	40	10
Results of actual inhibition zone diameters of colistin and fosfomycin disc diffusion testing				
	Inhibition zone size, mean ± S.D., mm		Inhibition zone size, mean ± S.D., mm	
Colistin	305	12.95 ± 2.87	27	14.77 ± 2.64
Fosfomycin	305	12.50 ± 4.63	27	23.19 ± 5.04

### Clinical characteristics and treatment outcomes of CRE-infected patients who received different types of best available antimicrobial therapy

Definitive antimicrobial treatment was categorized into four BAAT regimens; colistin monotherapy (CM), non-colistin monotherapy (NCM), colistin-based combination therapy (CCT), and non-colistin-based combination therapy (NCCT). Most baseline characteristics, including the Charlson comorbidity index, APACHE II score, medical procedures carried out before the onset, onset and site of CRE infection, and length of hospitalization, were comparable (Table 4). However, patients who received the NCM regimen had significantly lower rates of MV support (*p* = 0.001), placement of CVC (*p* < 0.001), and undergoing hemodialysis (*p* = 0.019) before developing CRE infections. Pneumonia as a cause of CRE infection (*p* = 0.025) and co-infection with other organisms (*p* < 0.001) occurred significantly less often than in those who received other regimens. At the onset of CRE infection, a group of patients who received the NCM regimen had lower SOFA scores (*p* < 0.001), less frequent septic shock (*p* < 0.001), and fewer deaths (*p* < 0.001).

**TABLE 4:** Comparison of clinical characteristics and treatment outcomes of carbapenem-resistant *Enterobacterales* -infected patients who received colistin monotherapy (CM) vs. colistin-based combination therapy (CCT) vs. non-colistin monotherapy (NCM) vs. non-colistin-based combination therapy (NCCT).

Characteristic	CM (109)	CCT (163)	NCM (47)	NCCT (45)	*P*-value^a^
Male, *n* (%)	55 (50.46)	92 (56.44)	24 (51.06)	28 (62.22)	0.519
Female, *n* (%)	54 (49.54)	71 (43.56)	23 (48.94)	17 (37.78)	0.519
Age, mean ± S.D., years	64.88 ± 20.97	63.87 ± 18.61	68.45 ± 18.16	66.78 ± 15.45	0.478
BMI, mean ± S.D., kg/m^2^	22.47 ± 4.94	23.39 ± 5.88	23.49 ± 4.15	22.03 ± 4.38	0.449
Underlying diseases, *n* (%)					
Hypertension	59 (54.13)	97 (59.51)	22 (46.81)	22 (48.89)	0.344
Diabetes mellitus	27 (24.77)	58 (35.58)	22 (46.81)	16 (35.56)	0.05
Chronic kidney disease	28 (25.69)	44 (26.99)	8 (17.02)	12 (26.67)	0.571
Cardiac diseases	23 (21.10)	35 (21.47)	8 (17.02)	6 (13.33)	0.611
Liver diseases	14 (12.84)	26 (15.95)	8 (17.02)	11 (24.44)	0.364
Cerebrovascular diseases	19 (17.43)	15 (9.20)	8 (17.02)	5 (11.11)	0.186
Chronic lung diseases	6 (5.50)	7 (4.29)	0	1 (2.22)	0.376
Solid malignancy	46 (42.20)	64 (39.26)	23 (48.94)	22 (48.89)	0.522
Hematologic malignancy	20 (18.35)	33 (20.25)	6 (12.77)	5 (11.11)	0.408
Receiving immunosuppressive agent	16 (14.68)	21 (12.88)	3 (6.38)	7 (15.56)	0.5
Autoimmune diseases	14 (12.84)	13 (7.98)	1 (2.13)	2 (4.44)	0.1
Post-organ transplantation	4 (3.67)	8 (4.91)	1 (2.13)	6 (13.33)	0.059
HIV infection	3 (2.8)	4 (2.52)	0	0	0.738
Cause of hospitalization, *n* (%)					
Infection-related	65 (59.63)	91 (55.83)	31 (65.96)	28 (62.22)	0.608
Charlson comorbidity index, mean ± S.D.	4.71 ± 2.43	4.64 ± 2.62	5.17 ± 2.70	5.5 ± 2.62	0.758
APACHE II score at admission, mean ± S.D.	13.89 ± 6.22	13.91 ± 6.45	13.74 ± 5.95	13.66 ± 6.40	0.913
Medical procedure before the onset of CRE infection, *n* (%)					
On mechanical ventilatory support	80 (73.39)	104 (63.80)	19 (40.43)	30 (66.67)	0.001^b^
Central venous catheter placement	63 (57.80)	89 (54.60)	10 (21.28)	24 (53.33)	<0.001^b^
On hemodialysis	39 (35.78)	59 (36.20)	6 (12.77)	14 (31.11)	0.019^b^
Major surgery	27 (24.77)	43 (26.38)	11 (23.40)	14 (31.11)	0.833
Receiving TPN	19 (17.43)	26 (15.95)	2 (4.26)	9 (20.00)	0.133
Receiving concurrent immunosuppressants	16 (14.68)	21 (12.88)	3 (6.38)	7 (15.56)	0.500
Tracheostomy	14 (12.84)	21 (12.88)	5 (10.64)	4 (8.89)	0.77
Percutaneous intervention	12 (11.01)	20 (12.27)	5 (10.64)	7 (15.56)	0.867
Concurrent chemotherapy	12 (11.01)	16 (9.82)	5 (10.64)	5 (11.11)	0.988
Bronchoscopy	12 (11.01)	18 (11.04)	4 (8.51)	1 (2.22)	0.318
ERCP	7 (6.42)	14 (8.59)	4 (8.51)	6 (13.33)	0.582
EGD	7 (6.42)	10 (6.13)	2 (4.26)	6 (13.33)	0.306
Colonoscopy	4 (3.67)	8 (4.91)	1 (2.13)	2 (4.44)	0.85
Minor surgery	3 (2.75)	3 (1.84)	1 (2.13)	1 (2.22)	0.968
Onset before developing CRE infection, mean ± S.D., days	26.98 ± 56.32	26.54 ± 31.28	14.81 ± 17.18	18.13 ± 18.49	0.165
Site of CRE infection, *n* (%)					
Bacteremia	30 (27.52)	55 (33.74)	10 (21.28)	15 (33.33)	0.344
Pneumonia	40 (36.70)	50 (30.67)	8 (17.02)	8 (17.78)	0.025^c^
Intraabdominal infection	19 (17.43)	35 (21.47)	15 (31.91)	12 (26.67)	0.207
Urinary tract infection	15 (13.76)	15 (9.20)	10 (21.28)	8 (17.78)	0.121
Others	5 (4.59)	8 (4.91)	4 (8.51)	2 (4.44)	0.701
SOFA score at onset, mean ± S.D.	7.17 ± 4.18	6.80 ± 3.77	4.91 ± 3.50	7.33 ± 4.44	<0.001^b^
Septic shock, *n* (%)	81 (74.31)	117 (71.78)	20 (42.55)	34 (75.56)	<0.001^b^
Co-infection with other bacteria, *n* (%)	75 (68.81)	106 (65.03)	23 (48.94)	31 (68.89)	<0.001^b^
Appropriate empiric treatment, *n* (%)	14 (12.84)	14 (8.59)	3 (6.38)	6 (13.33)	0.468
Outcomes					
Death, *n* (%)	76 (69.72)	108 (66.26)	17 (36.17)	31 (68.89)	<0.001^b^
Length of hospitalization, mean ± SD, days	47.4 ± 61.91	51.52 ± 49.75	38.79 ± 38.79	41.11 ± 37.35	0.384

a χ^2^ test and One-way ANOVA or Kruskal–Wallis test were used to compare the patient characteristics who received the different BAAT regimens.

b There is a statistical difference (*p* < 0.05) between CM vs. NCM, CCT vs. NCM, and NCCT vs. NCM.

c There is a statistical difference (*p* < 0.05) between CM vs. CCT and CM vs. NCCT.

APACHE II, acute physiological assessment and chronic health evaluation II; BAAT, best available antimicrobial therapy; CRE, carbapenem-resistant Enterobacterales; EGD, esophagogastroduodenoscopy; ERCP, endoscopic retrograde cholangiopancreatography; HIV, human immunodeficiency virus; SOFA, sequential organ failure assessment; TPN, total parenteral nutrition.

## Discussion

Our study demonstrated that carbapenem-resistant *Enterobacterales* cause substantial hospital-related clinical and financial burdens. Among 420 adult hospitalized patients with CRE infections, 380 (86.67%) were infected with carbapenem-resistant *K. pneumoniae*, one of the most common causes of CRE hospital-associated infections (de Maio Carrilho et al., 2016; Wang et al., 2016; Chotiprasitsakul et al., 2019). Most patients with CRE infection were elderly and had multiple comorbidities such as hypertension, malignancy, diabetes mellitus, and chronic kidney disease. Similarly, Wang et al. reported that patients with comorbidities have a higher rate of CRE infection (Wang et al., 2016). However, hypertension, diabetes mellitus, and malignancy are common medical conditions in the elderly, so a causal relationship between CRE infection and such comorbidities should not be assumed. In addition, we did not observe a difference in the CRE infection rate between men and women, in contrast to another study that reported a higher infection rate in men (Kang et al., 2019). Approximately 61% of patients were hospitalized due to infectious diseases, and 41% had a history of antimicrobial prescription in the preceding 3 months. Our findings were similar to several studies showing that patients who received broad-spectrum beta-lactams, such as carbapenems or cephalosporins, are at increased risk of developing CRE infection (Wang et al., 2016; Kang et al., 2019; Nicolas-Chanoine et al., 2019). Our CRE-infected patients had high APACHE II scores, critical illnesses requiring MV support (62.86%), and required placement of CVC (49.00%) before the onset. Likewise, two other studies concluded that patients infected by carbapenem-resistant strains were more likely to have respiratory compromise and unstable hemodynamics requiring MV support and CVC placement than those infected by carbapenem-susceptible strains (Wang et al., 2016; Zhang et al., 2021).

The onset of CRE infection varied from a few days to several weeks following hospital admission. Forty-five patients had been admitted to another hospital before transferring to our center. We could not precisely determine the onset of CRE infection in these patients because we did not know the duration of stay at the referring hospital. Bacteremia and pneumonia were the primary sites of CRE infection, while intraabdominal and urinary tract infections were less common. Other studies have reported that CRE bacteremia was found in only 7%–19% of patients, while pneumonia (41–57%) and urinary tract infection (UTI) (40%) were more common (Wang et al., 2016; Zhang et al., 2018; Zhang et al., 2021). Our subjects were mainly critically ill (40–60%) and required MV support or CVC placement, while the proportion of critically ill patients noted in other studies was only 20–40% (Zhang et al., 2018; Zhang et al., 2021). This likely increased the risk of CRE bacteremia and pneumonia in our study. Among patients with CRE infection, nearly 64% had co-infections with other multidrug-resistant (MDR) Gram-negative bacteria, such as *A. baumannii*, *P. aeruginosa*, *Enterobacter* spp., and *S. maltophilia*. These MDR bacteria are significant threats to HAI in hospitalized patients (Sirijatuphat et al., 2018; Jitmuang et al., 2020). In addition, HAI, such as pneumonia, intraabdominal infection, and UTI, are sometimes caused by polymicrobial infection (20%–30%) (Jitmuang et al., 2020; Liu et al., 2020; Sirijatuphat et al., 2020). Prolonged hospitalization, prior broad-spectrum antimicrobials administration, undergoing medical procedures, and severity of infection were associated with the acquisition of CRE and other MDR bacteria co-infections in our study. However, in the presence of multiple organisms, colonization and actual infection can be challenging to distinguish, and this may have resulted in an overestimation of co-infection rates.

An epidemiological study reported that *bla*NDM (63%), *bla*OXA-48-like (48%), and the coexistence of both genes (16%) are more prevalent carbapenemases in Thailand (Paveenkittiporn et al., 2021). Antimicrobials against CRE isolates with *bla*NDM carbapenemase are limited and lack sufficient clinical study (Kengkla et al., 2021). We compared the AST patterns of the CRE isolates in our study to previous and recent studies; it seems that the AST patterns were not changeable over time. CRE isolates are still moderately susceptible to aminoglycosides, such as amikacin (57–87%), gentamicin (31–67%), and netilmicin (34–65%). Meanwhile, they have increased resistance to fluoroquinolones, tetracyclines, and co-trimoxazole (Yungyuen et al., 2021; Nulsopapon et al., 2022). Therefore, our hospital’s best antimicrobials for treating CRE infection are colistin, fosfomycin, tigecycline, and aminoglycosides. However, concerns about potential toxicities in critically ill patients make many clinicians hesitant to prescribe those agents as an early empiric treatment before knowing if the isolate is confirmed CRE. Consequently, we have documented that approximately 50–90% of patients with CRE infection received inappropriate empiric antimicrobials (Kengkla et al., 2021). Colistin MICs determined by broth microdilution (BMD) method are used as reference susceptibility testing (Weinstein and Lewis, 2020), but colistin BMD is not available in our institute. However, our previous study showed that colistin D.D. using inhibition zone diameters moderately correlates with colistin susceptibility in carbapenem-resistant *E. coli* and *K. pneumoniae* isolates (Ruangkriengsin et al., 2018). Therefore, colistin was commonly administered as a single or an add-on agent in this study. Fosfomycin has good *in vitro* activity against CRE isolates and provides an additional effect when combined with other antimicrobials (Yu et al., 2017). A high-dose regimen of intravenous fosfomycin exhibited *in vitro* microbiological eradication in severe CRE infections (Kanchanasurakit et al., 2020). Tigecycline is generally recommended for treating intraabdominal infections as it has broad activity against several carbapenemases when using high doses or in combination with other agents (Ni et al., 2016; Durante-Mangoni et al., 2019). CRE isolates exhibit moderate to high susceptibility rates to aminoglycosides (Table 3). Due to the high concentration in urine, this agent is an alternative to treat CRE urinary tract infection when the isolate is susceptible (Amladi et al., 2019), but potential renal toxicity is a concern. Patients who received NCM were less severely ill before the onset of CRE infection and had fewer fatal outcomes. In contrast, patients that received other regimens, such as colistin- and non-colistin-based combination therapy, had more severe conditions and higher mortality. While colistin remains effective against CRE isolates (Amladi et al., 2019; Durante-Mangoni et al., 2019), and colistin combined with other agents has more killing activity (Zhou et al., 2019; Nutman et al., 2020), several clinical studies have demonstrated increased mortality and nephrotoxicity in colistin-based regimens compared to regimens with newer agents (van Duin et al., 2018; Wunderink et al., 2018; Motsch et al., 2020). Our findings suggest that in Thailand, where *bla*NDM is prevalent, and carbapenemase testing is not routinely performed, regimens containing colistin may not be the optimal choice. Newer antimicrobials to combat CRE isolates are urgently needed to improve favorable outcomes.

The overall mortality rate in our study was 68.33%, comparable to the 50–60% reported in other studies (Wang et al., 2016; Soontaros et al., 2022). Time to appropriate antimicrobial therapy was an independent predictor of mortality in bacteremic CRE infection (Falcone et al., 2020), but patients who received the appropriate empiric treatment in this study did not show a statistical difference between survival and non-survival outcomes (*p* = 0.719). Clinical features such as requiring MV support, high SOFA score, having septic shock at the onset, and having co-infection with other bacteria were strongly associated with in-hospital mortality. Meanwhile, appropriate definitive antimicrobial therapy did not protect against mortality, similar to findings from earlier studies (de Maio Carrilho et al., 2016; Zhang et al., 2021). CRE infection also increased hospital costs, mainly due to the requirement for more intensive medical care and prolonged hospitalization. Compared with other conditions, the hospital and economic burdens of CRE infection are more substantial than other MDR bacteria, such as *A. baumannii* (Lee et al., 2010; Nelson et al., 2016), vancomycin-resistant enterococci (Butler et al., 2010), and methicillin-resistant *Staphylococcus aureus* (Lodise and McKinnon, 2005). In addition, CRE infection causes more annual healthcare costs compared with other chronic diseases, such as hypertension, asthma, and diabetes (Bartsch et al., 2017). Therefore, CRE prevention and control strategies should be applied to reduce the burdens of CRE infection.

This study has some limitations. It was a retrospective study, and data were primarily obtained from medical chart review, so information on some variables was incomplete. We did not include a small group of terminally ill patients who received only the best supportive care in the outcome analysis, which may have introduced bias in comparing treatment outcomes. In-hospital all-cause mortality as the treatment outcome may have been influenced by other medical conditions unrelated to CRE infection. In some cases, multiple bacteria were isolated from the same specimen, so it was sometimes difficult to determine which one was the causative agent. In these cases, co-infection with multiple organisms was assumed. Antibiotic susceptibility testing of colistin and fosfomycin was not sufficiently standardized to represent their actual activities against the CRE isolates. As long as newer and more effective antimicrobials remain unavailable in Thailand, clinical outcomes based on the BAAT will continue to be unsatisfactory. Our results may not apply to different regions and settings. A prospective, well-designed, case-control study is needed to measure the burden of CRE infection more precisely.

## Conclusion

Carbapenem-resistant *Enterobacterales* cause substantial hospital-related clinical and financial burden. *In our study, K. pneumoniae* and *E. coli* were the most common carbapenem-resistant *Enterobacterales*. Hospitalized patients with CRE infection usually had a recent hospitalization, received broad-spectrum antimicrobials, or had undergone invasive medical procedures. Most infected patients had severe clinical parameters and septic shock at the onset. CRE infection led to high mortality rates, particularly in those who received MV support, had septic shock, had high SOFA scores, or had other bacterial co-infection. The best available antimicrobial therapy, such as colistin- and non-colistin-based regimens, resulted in high rates of adverse outcomes. Therefore, newer antimicrobial agents are urgently needed.

## Data Availability

The raw data supporting the conclusions of this article will be made available by the authors, without undue reservation.
