# Supplementary material for: The burden of carbapenem-resistant Enterobacterales infection in a large Thai tertiary care hospital
Source: Front Pharmacol. 2022 Sep 2;13:972900. doi: 10.3389/fphar.2022.972900 (PMC9479096; doi:10.3389/fphar.2022.972900)
Supplement: Supplementary file 1 [file Table1.docx]

**Supplementary Table 1**. The admission costs in 2015 to 2019 were inflated to 2021 Thai Baht using Thai medical care consumer price index (CPI) as displayed in the Table

| **Year** | **CPI (based 2019)** |
| --- | --- |
| 2015 | 0.9695 |
| 2016 | 0.9783 |
| 2017 | 0.9840 |
| 2018 | 0.9929 |
| 2019 | 1.0000 |
| 2020 | 1.0042 |
| 2021 | 1.0104 |

Reference: http://www.price.moc.go.th/price/cpi/index_new_all.asp

**Supplementary Table 2**. Comparison of clinical characteristics and management between survival and non-survival groups of patients who had carbapenem-resistant *Enterobacterales* infection

| **Characteristics** | **Non-survival group** (n=232)^a^ | **Survival group** (n=132)^a^ | ***P*-value** |
| --- | --- | --- | --- |
| Male, n (%) (199) | 122 (61.31) | 77 (38.69) | 0.325 |
| Female, n (%) (165) | 110 (66.67) | 55 (33.33) | 0.171 |
| Age, mean± S.D., years | 68.16±18.78 | 59.79±19.14 | < 0.001 |
| BMI, mean ± S.D., kg/m² (248) | 22.85±4.62 | 23.03±5.62 | 0.8 |
| **Underlying diseases**, n (%) |  |  |  |
| Hypertension (200) | 140 (70.00) | 60 (30.00) | 0.006 |
| Solid malignancy (155) | 92 (59.35) | 63 (40.65) | 0.152 |
| Diabetes mellitus (123) | 79 (64.23) | 44 (35.77) | 0.909 |
| Chronic kidney disease (92) | 65 (70.65) | 27 (29.35) | 0.132 |
| Cardiac diseases (72) | 50 (69.44) | 22 (30.56) | 0.277 |
| Liver diseases (59) | 35 (59.32) | 24 (40.68) | 0.462 |
| Hematologic malignancy (64) | 41 (64.06) | 23 (35.94) | 1 |
| Cerebrovascular diseases (47) | 31 (65.96) | 16 (34.04) | 0.871 |
| Receiving immunosuppressive agent (47) | 32 (68.09) | 15 (31.91) | 0.626 |
| Autoimmune diseases (30) | 22 (73.33) | 8 (26.67) | 0.323 |
| Chronic lung diseases (14) | 12 (85.71) | 2 (14.29) | 0.095 |
| HIV infection (8) | 5 (62.50) | 3 (37.50) | 1 |
| **Cause of hospitalization**, n (%) |  |  |  |
| Infection-related (215) | 139 (64.65) | 76 (35.35) | 0.74 |
| Non-infection related (149) | 93 (62.42) | 56 (37.58) | 0.74 |
| Charlson comorbidity index, mean± S.D. | 5.11±2.45 | 4.34±2.72 | 0.004 |
| APACHE II score at admission, mean± SD | 14.86± 6.52 | 12.06±5.44 | <0.001 |
| **Past medical history**, n (%) |  |  |  |
| Previous antimicrobial administration in last 3 months (144) | 96 (66.67) | 48 (33.33) | 0.374 |
| Previous hospitalization in last 3 months (171) | 113 (66.08) | 58 (33.92) | 0.385 |
| Prior CRE colonization^b^ (35) | 28 (80.00) | 7 (20.00) | 0.041 |
| Previous surgery within 1 month (18) | 9 (50.00) | 9 (50.00) | 0.219 |
| **Medical procedure before the onset of CRE infection**, n (%) | | | |
| On mechanical ventilatory support (233) | 186 (79.83) | 47 (20.17) | <0.001 |
| Central venous catheter placement (186) | 146 (78.49) | 40 (21.51) | <0.001 |
| On hemodialysis (118) | 96 (81.36) | 22 (18.64) | <0.001 |
| Major surgery (95) | 54 (56.84) | 41 (43.16) | 0.108 |
| Receiving TPN (56) | 43 (46.79) | 13 (23.21) | 0.034 |
| Receiving concurrent immunosuppressants (47) | 32 (68.09) | 15 (31.91) | 0.626 |
| Tracheostomy (44) | 32 (72.73) | 12 (27.27) | 0.242 |
| Percutaneous intervention (44) | 22 (50.00) | 22 (50.00) | 0.047 |
| Concurrent chemotherapy (38) | 16 (24.11) | 22 (57.89) | 0.429 |
| Bronchoscopy (35) | 23 (65.71) | 12 (34.29) | 0.855 |
| ERCP (31) | 14 (45.16) | 17 (54.84) | 0.031 |
| EGD (25) | 19 (76.00) | 6 (24.00) | 0.205 |
| Colonoscopy (15) | 12 (80.00) | 3 (20.00) | 0.273 |
| Minor surgery (8) | 2 (25.00) | 6 (75.00) | 0.029 |
| Onset before developing CRE infection, median (IQR), days | 19 (9, 31) | 11 (3, 22) | <0.001 |
| **Site of CRE infection**, n (%) |  |  |  |
| Bacteremia (110) | 73 (66.36) | 37 (33.64) | 0.493 |
| Pneumonia (106) | 89 (83.96) | 17 (16.04) | <0.001 |
| Intraabdominal infection (81) | 37 (45.68) | 44 (54.32) | <0.001 |
| Urinary tract infection (48) | 20 (41.67) | 28 (58.33) | <0.001 |
| Others (19) | 13 (68.42) | 6 (31.58) | 0.663 |
| SOFA score at onset, median (IQR) (364) | 8.00 (5, 11) | 4.00 (2, 6) | <0.001 |
| Septic shock, n (%) (364) | 211 (83.73) | 41 (16.27) | <0.001 |
| Co-infection with other bacteria, n (%) (235) | 175 (74.47) | 60 (25.53) | <0.001 |
| **Antimicrobial administration**, n (%) |  |  |  |
| Appropriate empiric treatment (37) | 25 (67.57) | 12 (32.43) | 0.719 |
| **Best available antimicrobial therapy (BAAT)** |  |  |  |
| Antimicrobial monotherapy, n (%) (156) | 93 (59.62) | 63 (40.38) | 0.186 |
| Colistin monotherapy (109) | 76 (69.72) | 33 (30.28) | 0.598 |
| Non-colistin monotherapy (47) | 17 (36.17) | 30 (63.82) | 0.002 |
| Antimicrobial combination therapy, n (%) (208) | 135 (64.90) | 69 (33.17%) | 0.186 |
| Colistin-based combination therapy (163) | 108 (66.26) | 55 (33.74) | 0.598 |
| Non-colistin-based combination therapy (45) | 31 (68.89) | 14 (31.11) | 0.002 |

Abbreviations: APACHE II, acute physiological assessment and chronic health evaluation II; BAAT, best available antimicrobial therapy; CRE, carbapenem-resistant *Enterobacterales*; EGD, esophagogastroduodenoscopy; ERCP, endoscopic retrograde cholangiopancreatography; HIV, human immunodeficiency virus; SOFA, sequential organ failure assessment; TPN, total parenteral nutrition

^a^ Excluded 56 patients who received only best supportive care with no antimicrobial therapy

^b^ Defined by cultures positive with no clinical infection and no antimicrobial therapy
